# Recalibrating Risk: A Call for South Asian-Focused Heart Failure Prediction Models

**DOI:** 10.3390/jcm15166181

**Published:** 2026-08-10

**Authors:** Nandini Nair, Dongping Du, Aiswarya J. Pillai, Swarna Mahesh, Mrudula R. Munagala, Howard J. Eisen, Balakrishnan Mahesh

**Affiliations:** 1 Division of Cardiology, Heart and Vascular Institute, Penn State Health Milton S. Hershey Medical Center, Hershey, PA 17033, USAsmahesh555000@gmail.com (S.M.); 2 Department of Industrial, Manufacturing and Structural Engineering, Texas Tech University, Lubbock, TX 79409, USA; 3 Division of Cardiology, Miller School of Medicine, University of Miami, Miami, FL 33101, USA; mrm410@med.miami.edu; 4 Division of Cardiology, Thomas Jefferson University, Philadelphia, PA 19107, USA; howardeisen56@gmail.com; 5 Division of Cardiothoracic Surgery, Heart and Vascular Institute, Penn State Health Milton S. Hershey Medical Center, Hershey, PA 17033, USA; drbmahesh@gmail.com

**Keywords:** risk assessment, heart failure, South Asian Indians, Indian diaspora, heart failure in Indians, genetic determinants of heart disease in Indians, dyslipidemia-related risk in South Asian Indians

## Abstract

**Background**: Heart failure (HF) represents the final common pathway of diverse cardiovascular disorders, including coronary artery disease, primary myocardial pathology, and abnormalities of cardiac conduction. These conditions arise from an interplay of genetic, environmental, and psychosocial influences, making it essential to understand these determinants to improve risk prediction and prevention. Multiple biological pathways of inflammation, fibrosis, coagulation, oxidative stress, lipid dysregulation, endothelial dysfunction, and metabolic disturbances are shaped by inherited susceptibility and modifiable exposures. Together, these mechanisms drive HF development and progression, though their relative contributions vary across populations. **Methods**: PubMed and Google Scholar were searched for clinical, biomedical, and interdisciplinary studies published between 1 January 2000, and 31 December 2025. Keywords included “Asian,” “adult,” “India,” “heart failure,” “risk assessment,” “prognosis,” and “predictive value.” Studies were included if they focused on South Asian Indian adults, with priority given to original research, systematic reviews, and meta-analyses. Pediatric studies and those centered on other ethnic groups were excluded. **Results**: Among South Asian Indians, cardiovascular disease burden remains disproportionately high compared with Western populations. Unique genetic architecture, environmental exposures, and sociocultural factors appear to contribute to earlier onset and more aggressive disease. Identifying population-specific genetic variants, clarifying psychosocial influences, and addressing environmental risks may help reduce these disparities. **Conclusions**: This qualitative review highlights key gaps in current knowledge. A deeper understanding of these determinants could refine HF risk stratification, guide targeted prevention strategies, and reduce the growing cardiovascular burden in South Asian Indians. There is an urgent need for South Asian specific HF risk prediction models.

## 1. Introduction

Heart failure (HF) is a global epidemic, with a current prevalence of approximately 40 million patients and an estimated rise of 25% by the year 2030 [1,2,3]. Though the prevalence of HF increases with age, in India, the age of onset appears to be at least a decade lower than in other countries [4,5]. Indian patients have a higher prevalence of coronary artery disease (CAD), hypertension, and diabetes influenced by diet, lifestyle and genetic determinants. Additionally, important differences exist between different socioeconomic groups within the same race and ethnicity. HF in Asia carries different phenotypes, suggesting that in different countries HF will need specifically tailored measures to prevent and treat the disease to improve outcomes [2,6]. As per World Health Organization (WHO) statistics, the highest number of deaths due to HF occurs in India as compared to the rest of the world. In 2017, 1.7 million deaths were due to HF in India, and this is expected to increase to 2.2 million by the year 2030. This review attempts to address the risk enhancers, risk factors and role of ethnic and genetic determinants for cardiovascular disease and the end result of heart failure in this sub population of Asia to identify strategies for improved outcomes. The review highlights the dearth of definitive clinical trials addressing HF in South Asian Indians.

## 2. Materials and Methods

PubMed and Google Scholar were searched to identify relevant clinical, biomedical, and interdisciplinary studies published between 1 January 2000, and 31 December 2025. The search included keywords such as “Asian,” “adult,” “India,” “heart failure,” “risk assessment,” “prognosis,” and “predictive value.” Studies were included if they focused on adults (≥18 years) of South Asian Indian origin, with priority given to original research articles, systematic reviews, and meta-analyses. Studies involving pediatric or adolescent populations, as well as those focusing on other ethnic groups, were excluded.

**Figure 1:**
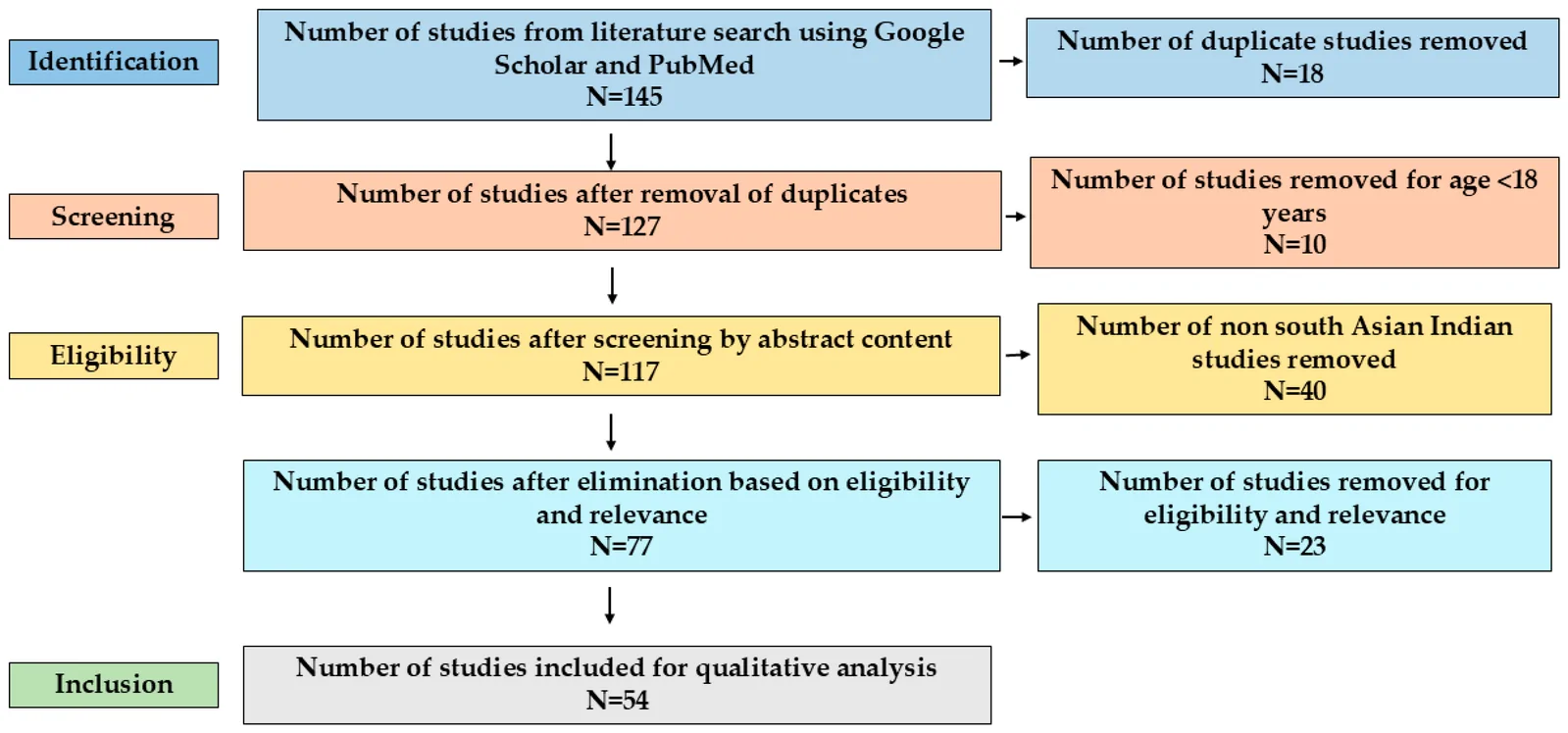
PRISMA-style flow diagram used to document the study selection process.

To ensure the quality of the data, studies with unclear or incomplete data, non-peer-reviewed publications, and those published outside the specified date range were excluded. The screening process was conducted in two stages: an initial review of titles and abstracts, followed by full-text evaluation of potentially relevant articles. A PRISMA-style flow diagram was used to document the study selection process (Figure 1).

A total of 145 articles were initially identified. After removing 18 duplicates, further exclusions included 10 pediatric studies, 40 studies not involving South Asian populations, and 23 studies deemed not relevant to the research question. This resulted in a final set of 54 studies included in the analysis.

### 2.1. Epidemiology

Given the lack of a completed national registry, the reported incidence of HF varies widely in India from 1.3 to 23 million. The distribution of HF with reduced ejection fraction (HFrEF) and HF with preserved ejection fraction (HFpEF) is also highly variable in the existing individual registries. It is interesting that the HFpEF population ranges from 0.4% to 25% in the different registries. It is unclear whether this is a matter of missing data or regional variations within the Indian subcontinent [2,6,7,8,9,10,11]. The current prevalence of HF can be estimated to be approximately 10 million in India [5].

### 2.2. Risk Enhancers

Heart failure is the end point of diverse cardiac diseases. Genetic and psychosocial factors shape inflammation, fibrosis, metabolism, and vascular dysfunction. Understanding these determinants and modifying the environmental risks may improve the prediction and help reduce the disproportionate cardiovascular burden in South Asian Indians as compared to other races [12].

#### 2.2.1. Genetic Determinants

A higher prevalence of cardiovascular disease and a higher risk of cardiovascular mortality exist in India as compared to other global populations. Changes in lifestyles due to urbanization and evolving cultural, socioeconomic and environmental factors may in part be responsible for this effect. However, the existing methods of cardiovascular risk prediction remain based on Caucasian populations. Current data support a strong contribution of genetic factors in different races. Genome-Wide Association Studies (GWAS) for genetic and functional studies are required to identify genetic loci and elucidate the influence of associated loci on the cardiovascular risk profile. Prospective studies are needed to shed light on the actual cardiovascular morbidity and mortality rates to compare the actual and predicted rates.

Differences in the prevalence of metabolic risk factors and CVD between South Asians and non-South Asian populations may be driven in part by differences in genetic variation. The existing data suggest that genetic factors contribute to cardiac diseases and predispose individuals, generating disparity in different races. Genetic predisposition to diabetes, dyslipidemias and cardiovascular diseases have been established in different races in the past [13]. Family history suggests a genetic predisposition in addition to environmental and cultural factors associated with CVD. The family history of ischemic heart disease predicts a 2.5- to 7-fold increase in mortality. However, it is also important to note that variations exist in genetic determinants even within a race, and such variations should be considered in developing diagnostic and therapeutic strategies that are race-based [14]. In the absence of genome-wide association studies in Indians and other non-Caucasian races, the precise estimation of the heritability of relevant genetic loci in the different ethnic groups still remains difficult [15,16].

Genetic studies in the South Asian diaspora living in different parts of the world may help to partially decipher the genetic predisposition to cardiovascular studies in the population living in India, though environmental factors may exert a differential effect epigenetically in the different parts of the globe. GWAS, whole exome and genomic studies in the general population will help elucidate the differences in the genetic framework for cardiovascular disease risk factors such as diabetes, obesity and dyslipidemias in the South Asian population. Genetic variants for DM in the South Asian population identified at 6 new loci were GRB14, ST6GAL1, VPS26A, HMG20A, AP3S2, and HNF4A [17,18]. GWAS of DM in Punjabi Sikhs from India also found novel associations [18]. In South Asians, there appears to be no significant evidence for definite genetic determinants to explain central obesity [19]. Complications secondary to diabetes arise due to sustained interaction between the advanced glycation end products (AGEs) and the receptors for AGE (RAGE), which trigger detrimental signaling. Genetic variations in the RAGE gene may be associated with varying vascular complications in DM patients [20].

A variety of efforts are underway to better understand the population variation in India. Notably the Indian Genome Variation Project and the Indigenomes project are large-scale initiatives for datamining of whole genomes and exomes to better define genetic variation in a race which constitutes >17% of the world population. The extensive genetic diversity remains underrepresented in global sequencing projects. The Indian Genome Variation Consortium (IGVC) project supported by the Council for Scientific and Industrial Research (CSIR) has been the first large-scale comprehensive study of the Indian population. The aim of this project is to define genetic variants responsible for diseases and drug response to help develop diagnostic/therapeutic targets. Such an effort would be time consuming, especially in the setting of diverse sub populations in India. As part of the first phase, the IGV Browser has been generated to provide data on gene/allele frequency [21,22]. A multi-ancestry GWAS meta-analysis in 521,612 individuals (67,162 cases and 454,450 controls) showed 22 new stroke risk loci, with shared genetic variation with related vascular abnormalities, including blood pressure, cardiovascular diseases, and venous thromboembolism, at individual loci using genetic risk scores and linkage-disequilibrium-score regression. Several loci were associated with pleiotropic patterns for stroke with etiologically different subtypes. The analysis showed new mechanisms of stroke pathophysiology, which could open new avenues for drug therapy targets [23]. ClinIndb is a database constructed and linked to the Leiden Open Variation Database, which could be useful as it only represents the Indian sub populations. Further studies are required using such databases for developing genetically relevant screening tools as well as tailoring drug targets appropriate for the Indian population as well as the Indian diaspora throughout the world [24,25].

#### 2.2.2. Social Determinants

The socioeconomic status, level of education, clean air and water availability, the use of lead fuel and environmental pollution, nutrition/diet variation in different parts of the country and lack of supportive relationships free of discrimination and violence all contribute to heart disease leading to HF as the end point. Social determinants play a stronger role in susceptible populations.

#### 2.2.3. Conditions Specific to the Female Gender

Women can have specific risks such as chemo-related cardiotoxicity secondary to breast cancer treatment strategies, hormonal changes due to menopause, gestational diabetes, pre-eclampsia and post-partum cardiomyopathy (PPCM), all of which could lead to chronic systolic HF. Identification of the risk factors using deep learning algorithms applied to well defined registries will shed light on genetic- and gender-based risk attributes that would predispose women of different races/ethnicity to chronic HF.

#### 2.2.4. Dyslipidemia-Related Risk

The general patterns of disease progression/morbidity and mortality illustrate a predominance of ischemic heart disease in young individuals particularly <40 years of age. In India, cardiovascular disease is responsible for 24% of all deaths in patients in the age group of 25–69 years. Ten percent of all myocardial infarctions occur in patients <40 years. More than half of the mortality in patients <70 years of age appears to be due to cardiac disease, with disability-adjusted life-years (DALY) secondary to ischemia 3-fold higher in India as compared to the western nations.

Lipid profiles that are markedly abnormal with persistently elevated low density lipoproteins/triglycerides are risk enhancers for ischemic heart disease, eventually leading to ischemic cardiomyopathy and end stage HF if under diagnosed and undertreated.

#### 2.2.5. Concurrent Pro-Inflammatory Conditions

Increased inflammation is manifested by chronic diseases including peripheral arterial disease, rheumatoid arthritis, collagen vascular diseases (systemic lupus erythematosus (SLE), vasculitis, ankylosing spondylitis, chronic hepatitis C/Human Immunodeficiency Virus (HIV) infections, and metabolic syndrome, as well as chronic renal disease with an eGFR of 1–69 mL/min/1.73 m^2^, which can all be significant risk enhancers if not treated appropriately. Biomarkers of inflammation such C reactive Protein (CRP) > 2 mg/L, lipoprotein a >50 mg/mL and Apolipoprotein B levels >130 mg/dL can be windows to the impending pathophysiological doom [26,27].

### 2.3. Primary Prevention Strategies

#### 2.3.1. Risk Factor Modifications by Behavioral Interventions

Table 1 shows a variety of studies on South Asians to improve knowledge of cardiovascular risk and outcomes and lists those involving the modification of risk factors using lifestyle interventions. The list is by no means all-inclusive. However, none of the studies are directed toward HF patients. The studies in the existing literature are all directed toward risk factors that contribute to HF.

The SAHELI study showed that lifestyle interventions showed a small decrease in body weight and hemoglobin A1c levels in a pilot study of 68 patients. However, in the larger trial of 549 patients with cardiovascular risk factors with a 16-week culturally tailored diet plus physical activity and behavior programs in group sessions and follow-up support as compared to written health education, there were no significant improvements in blood pressure, lipids, HbA1c or weight. Improved self-reported diet and physical activity behaviors were noted in this study. These findings suggest that lifestyle programs change behavior, but translation into measurable cardiovascular risk reduction remains challenging in this population [27]. The Mediators of Atherosclerosis in South Asians Living in America (MASALA) study is a large, ongoing, community-based cohort designed to better understand why South Asians in the United States face a disproportionately high burden of cardiovascular disease. The study has enrolled more than 2300 participants and follows them over time, collecting detailed information on cardiometabolic risk factors, lifestyle behaviors, and imaging markers of early atherosclerosis. Findings from MASALA consistently show that South Asians have a high prevalence of conditions such as diabetes, hypertension, and dyslipidemia. In some subgroups, diabetes prevalence reaches 33%, obesity up to 48%, and hypertension up to 54%, highlighting the substantial burden of risk in this population. Importantly, these risk factors tend to appear earlier in life and at lower body mass index levels than seen in other populations. Additionally, approximately 61% of participants are classified as having an intermediate or high predicted 10-year risk for heart failure, underscoring the early and significant cardiovascular risk in this group. The MASALA study has also shed light on unique biological features, including increased visceral and ectopic fat and higher insulin resistance, which help explain why traditional measures of obesity may underestimate the risk in South Asians. Imaging findings further reveal a substantial burden of subclinical atherosclerosis, suggesting that commonly used cardiovascular risk prediction tools may not fully capture risk in this population. In summary these findings emphasize the need for earlier screening, more accurate risk assessment, and culturally tailored prevention strategies to reduce cardiovascular disease among South Asians. The MASALA study, which began in 2010, has resulted in >100 publications on the various aspects of risk factors and their prevention in South Asians [28]. One of the interesting aspects addressed from the MASALA cohort includes the prevalence of a higher calcium score in South Asians [29].

**Table 1:** Summary of selected studies on cardiovascular and metabolic risk in South Asians.

Study/Citation	Study Type	Sample Size	Arms	Intervention	Duration	Outcomes	Risk Metrics	Key Findings
**Kandula et al. (2024) [27]** [SAHELI *]	Randomized Control Pilot Study (Prospective)	N = 549; randomized 1:1 275 Intervention; 274 Control US-based South Asians in Chicago, IL	2 arms: Intervention vs. Control	Sixteen-week group lifestyle program coaching in 4 languages, delivered in person and via videoconference. Controls received monthly written materials. Participants tracked diet and physical activity with 4 optional follow-up sessions.	12 months	Primary: HbA1c, systolic/diastolic BP, weight, total cholesterol Secondary: diet quality, physical activity	No HR/RR/OR/NRI or HF-specific risk metrics reported. Mean differences in CV risk (weight, SBP, DBP, HbA1c, cholesterol) were not statistically significant.	Lifestyle interventions in community settings were not more effective than health education.
**Kanaya et al. (2014) [29]** **[MASALA subgroup]**	Cross-Sectional Study	Controls: 803 asymptomatic South Asians (India, Nepal, Pakistan, Sri Lanka) With cardiac disease: 2622 Whites; 1893 African Americans; 1496 Latinos; 803 Chinese Americans All South Asians living in the US	Cross-sectional comparison across ethnic groups	CAC Score determination in South Asians with/without cardiac disease to identify prevalence differences across ethnic groups.	Cross-sectional	Coronary Artery Calcium (CAC) Score determination across ethnic groups	S Asian vs. White males: RR = 0.92 (0.80–1.05) African American vs. S. Asian males: RR = 0.66 (0.58–0.76) Latino vs. S Asian males: RR = 0.77 (0.67–0.89) Chinese American vs. S. Asian males: RR = 0.83 (0.71–0.97) Association between race/ethnicity and age noted.	Higher CAC scores in South Asian and White males vs. Latinos or Chinese Americans. South Asian Women >70 had higher calcium scores than women of other ethnic groups.
**Muilwijk M et al. (2021) [30]** **[iHealth-T2D]**	Cluster Randomized Control Trial	N = 3684 total (1846 control; 1838 intervention) South Asians from India, Pakistan, Sri Lanka, UK; men and women 40–70 yrs with elevated HbA1c and waist circumference	2 arms: Family intervention (60 sites) vs. Usual care (60 sites)	Family-based lifestyle interventions: 5 individual sessions, 4 group and 13 telephone sessions. Goal: 7% BMI reduction and 5 cm waist decrease via diet and 150 min/wk of physical activity. Usual care: one 30 min lifestyle session. All sessions delivered by community health workers.	12 months	Weight, waist circumference, blood pressure, HbA1c	Weight: intervention −1.8 kg vs. control −0.4 kg; AMD −1.1 kg (95% CI −1.70 to −1.06), *p* < 0.001 Waist circumference: AMD −1.9 cm (95% CI −2.5 to 1.3), *p* < 0.001 BP and HbA1c: not significant	Weight and waist circumference are modifiable risk factors for T2DM. Blood pressure and HbA1c improvements were not significant.
**Tandon et al. (2022) [31]** **[LIVING Trial]**	Randomized Control Trial (women with gestational diabetes)	N = 1601 (Control = 801; Intervention = 800) South Asian women (India, Bangladesh, Sri Lanka) with gestational DM from urban centers	2 arms: Lifestyle intervention vs. Usual care	Four 90 min face-to-face group sessions over 6 months + 2 individual sessions for persistently overweight participants. Focus on diet and physical activity. Controls received no instructions. COVID-19 pandemic influenced delivery.	12 months	Primary: time to change in glycemic status Secondary: new-onset T2DM, change in body weight	HR = 0.92 (95% CI 0.76–1.12); *p* = 0.42—did not reduce worsening glycemic status. No improvement in any secondary outcome. No HF-specific metrics reported.	Interventional care did not prevent diabetes in women with prior gestational DM.
**Vlaar et al. (2017) [32]** **[South Asian Dutch Study]**	Randomized Control Trial (South Asians at risk for diabetes, Netherlands)	N = 536 enrolled (intervention = 283; control = 253) Analyzed: 314 participants (intervention = 165; control = 149); dropout noted South Asian immigrants from Uttar Pradesh, Uttaranchal, West Bihar via Suriname to Netherlands	2 arms: Culturally tailored program vs. Standard advice	Culturally tailored multicomponent program: motivational interviewing (6–8 sessions + 3–4 booster sessions), family session, cooking classes, supervised physical activity. Control received standard non-tailored lifestyle advice.	24 months	Physical activity, diet, and social-cognitive determinants at 2-year follow-up (independent *t*-tests, chi-square, Fisher’s exact tests)	Increase in moderate-to-vigorous physical activity in intervention but did not differ significantly from control (*p* = 0.672). No HF-specific metrics reported.	Culturally targeted lifestyle intervention did not lead to healthy behaviors in South Asians at high risk for diabetes.
**Bhopal et al. (2014) [33]** **[PODOSA Trial]**	Family Cluster Randomized Non-Blinded Clinical Trial (Indian/Pakistani adults at risk for T2DM, UK)	N = 171 (78 families; 85 intervention; 86 control) South Asians in UK; randomized by computer-generated sequence stratified by location, ethnicity, and family size	Two arms: 15 dietitian visits (intervention) vs. 4 visits (control)	Intervention: 15 dietitian visits over 3 years. Control: 4 visits. Primary outcome was 3-year weight change via modified linear regression.	36 months	Primary: incidence of diabetes Secondary: waist circumference, physical activity, insulin resistance, BP, fasting glucose	Diabetes progression: OR = 0.68 (*p* = 0.37, NS) Weight loss AMD = −1.64 (−2.83 to −0.44), *p* = 0.0076 BMI AMD = −0.60 (−1.06 to −0.14), *p* = 0.0112 Waist circ. AMD = −1.89 (−3.27 to −0.52), *p* = 0.0072 Waist:hip AMD = −1.54 (−2.71 to −0.37), *p* = 0.0101 Hip circ. AMD = −0.00 (−0.01 to 0.01), *p* = 0.68 (NS)	Modest weight loss achieved; no significant reduction in diabetes incidence. Insulin resistance parameters and BP did not differ significantly.
**Vahabi et al. (2015) [34]**	Community-Based Cohort Study (South Asian immigrant women, Greater Toronto Area) Combined descriptive cohort, pretest–posttest design and qualitative methods	N = 27 women (Indians, Sri Lankans, Bangladeshi, Nepalese, Pakistani)	Single arm (pre-post design)	Bollywood dance program led by a female instructor, 2 days/week × 6 weeks. Each 1 h session: 5 min warm-up, 45 min dance, 10 min cool-down.	6 weeks	Weight, BMI, waist and hip circumference monitored pre/post; social and mental health effects assessed	No HF-specific metrics reported. Weight, waist/hip circumference and BMI decreased but were not statistically significant.	Women found the program to be a stress reliever but did not associate it with health benefits.
**George J et al. (2017) [35]** **[CALIBER Registry]**	Registry-Based Cohort Study (CALIBER Registry)	N = 1,068,318 (90.9% White; 3.6% South Asian; 2.9% Black)	Registry-based ethnic group comparison (no intervention)	Used electronic health records from primary care, hospital admissions, ACS registry, and mortality registry (CALIBER) to study incidence of MI, stroke, and heart failure across ethnic groups.	5.7 years (mean follow-up)	Initial lifetime presentation of Heart failure Myocardial infarction	Heart Failure: Black HR = 0.97 (0.79–1.20); S Asian HR = 1.04 (0.87–1.26) Myocardial Infarction: Black HR = 0.49 (0.40–0.62); S Asian HR = 1.67 (1.49–1.87)	No ethnic differences in HF presentation. Initial MI presentation was significantly higher in South Asians and lower in Black patients compared to White patients.
**Agrawal et al. (2014) [36]**	Cross-Sectional Study (fish consumption and diabetes in adult Indians)	N = 156,316 (99,574 women; 56,742 men, aged 20–49 years, India)	Cross-sectional (no intervention); fish consumption frequency groups: daily, weekly, occasional, never	Multivariable-adjusted models examining associations between fish consumption frequency and self-reported diabetes, accounting for dietary exposures, BMI, smoking, alcohol, TV viewing, age, education, living standard, and location.	Cross-sectional	Association between fish consumption frequency and diabetes	Daily fish intake: OR = 2.02 (*p* < 0.0001) Weekly fish intake: OR = 1.55 (*p* < 0.0001) Stronger in men: OR = 2.46 Cross-spousal sensitivity tests: not significant	Daily or weekly fish consumption was positively associated with having diabetes, with stronger effects in men than women.
**Samaan et al. (2013) [37]**	Randomized Control Trial (Multimedia intervention to reduce CV risk)	Pilot study: N = 30 (Control = 15; Intervention = 15) Recruited from places of worship and community centers, Southwestern Ontario, Canada	Two arms: digital program vs. risk assessment only	Digital program: biweekly goal-setting, daily health messages, reminders, updated health info, healthy living videos to support diet, physical activity, smoking reduction, and reduced sedentary behavior. Risk score included genetic predisposition. Controls received risk assessment and were instructed to discuss with their family physician.	6 months	Diet quality, physical activity, smoking	No HF-specific risk metrics reported.	No statistically significant change between groups. Participants were motivated by risk scores, but not all multimedia components could be used to induce positive health behavior changes.

* SAHELI = South Asian Heart Lifestyle Intervention. LVEF was excluded from this table as none of the included studies documented this parameter. AMD = adjusted mean difference; BP = blood pressure; CAC = coronary artery calcium; CI = confidence interval; CV = cardiovascular; DM = diabetes mellitus; HbA1c = glycated hemoglobin; HF = heart failure; HR = hazard ratio; MI = myocardial infarction; NS = not significant; OR = odds ratio; RR = relative risk; SBP = systolic blood pressure; T2DM = type 2 diabetes mellitus.

The iHealth-T2D cluster randomized clinical trial, which was a family-based lifestyle program, designed to be practical and low-cost, helped participants lose weight and reduce their waist size over 12 months. Simple weight measurements, diet, and community health worker visits with phone support can lower the risk of developing type 2 diabetes in the long run. The study also found that people who attended more sessions saw more benefits, including better improvements in waist size, blood pressure, and HbA1c levels, highlighting the role of family-based culturally adapted interventions [29]. In the Living trial, in South Asian women with gestational diabetes, who had dysglycemia immediately after pregnancy, lifestyle interventions did not prevent glycemic deterioration, suggesting that additional approaches are needed in such high-risk populations [31]. Vlaar et al., in the South Asian Dutch study, found that even a strongly culturally tailored lifestyle program struggled to make a real impact. Despite being carefully designed for the target population, the intervention saw high dropout rates and did not significantly improve healthy behaviors among South Asians at risk for diabetes. Given this already elevated risk, there is a clear need to rethink our approach—focusing on strategies that feel more acceptable, engaging, and sustainable for this community in order to better support lasting lifestyle change [32]. Wallia et al., in the PODOSA trial, showed that there was only a modest weight loss with no significant reduction in diabetes incidence. In this trial, there were 15 home-based visits by dietitians over 3 years, but the trial was underpowered for assessing reduction in diabetes. The intervention was culturally tailored for language, diet, family structure and home delivery. The trial was meant to have a larger sample of >500 patients but could only enroll 171 patients, highlighting the challenges of recruiting minority populations, as the trial was conducted in Edinburgh and Glasgow. The trial also required multiple home visits by dietitians (15 visits), which raises concerns about the scalability and cost-effectiveness [33]. Vahabi and Damba showed that 2 days per week of physical exercise produced a significant reduction in body weight, waist/hip dimensions and body mass index) in 6 weeks, making culturally targeted gender-based exercises a viable option to improve risk management [34]. A study by George et al. showed South Asian patients were far more likely to present with coronary heart disease, while Black patients more often presented with stroke. However, no ethnic differences in how patients presented with heart failure were noted [35]. Agrawal et al. studied the effects of fish intake on diabetic Indians [36]. The SAHARA trial showed that multimedia approaches can instill positive behaviors influencing health risks, but these did not show statistically significant changes [37].

#### 2.3.2. Tobacco Use

Complete cessation of tobacco use/abuse by promoting public awareness is a key aspect. The pathophysiological effects of tobacco on cardiovascular disease initiation and progression clearly call for public education to stop abuse. Tobacco accelerates the development of atherosclerotic plaques composed of lipids/inflammatory substances and their disruption superimposed with formation of thrombi. Endothelial dysfunction is promoted by smoking and exposure to second-hand smoke, and the fine balance between levels of nitric oxide and endothelin that regulates endothelial function is lost. Increased oxidative stress via free radical generation, decreased nitric oxide synthesis and bioavailability and upregulation of inflammatory and prothrombotic mechanism will accelerate the initiation and progression of atherogenesis with adverse effect at all levels such as proteome, genome, epigenome, microbiome as well as the circadian rhythm. In addition, smoking cessation significantly reduces the risk of incidence of HF. More recent studies show that HF risk persists for a few decades after complete smoking cessation. Smoking is a risk factor for HF that can be modified by early intervention to prevent the incidence of HFrEF as well as HFpEF [38].

Tobacco use in India can occur in several different forms in addition to smoking. Several smokeless tobacco products flood the local markets, giving the consumer a wide variety of choices. The consumers’ tobacco choices are largely influenced by regional, demographic, social, economic and cultural practices. Predominantly smokers use a traditional tobacco hand-rolled and wrapped in dried tree leaves called a “bidi”, followed by the usual cigarettes. Additionally, a number of traditional hand-made products such as cigars, tobacco rolled in maize leaves or newspapers, tobacco in hukkah pipes called “chillum” and “chutta” also enter the market. Smokeless tobacco includes “khaini” a form of chewing tobacco made from sun-dried or fermented crushed tobacco leaves, “pan” (mixture of lime, areca nut pieces, tobacco and spices wrapped in betel leaf), “gutkha”, a powdered mixture of scented tobacco, lime and areca nut, and “mishri” which is a paste for rubbing on the gums. All forms of tobacco are detrimental to cardiac health [39,40,41]. Though some studies show a decline in tobacco use from 2009 to 2016 in order to achieve the National Health Policy aim of reducing tobacco consumption up to 15% by 2020 and up to 30% by 2025, targeted policies and interventions addressing socioeconomic inequalities must be implemented, such as sharp increases in the tobacco sales tax and the banning of smoking in all public places including transportation vehicles, and education regarding tobacco as a health hazard should be disseminated in all regional languages and local dialects to ensure the uniformity of distribution of important health information. This is important because there appears to be a higher prevalence of smokeless tobacco use in the northeastern states of India [42].

The 25–44-year-old age group showed a decline in smokeless tobacco use. The older age group showed a reluctancy to reduce smokeless tobacco use with increasing age, along with an increase in smokeless tobacco usage with age. This could be due to the addictive quality of tobacco. Tobacco control/cessation of the use of all smokeless tobacco product driven policies should target adolescents and younger age groups by improving the knowledge and awareness of the ill effects of tobacco to elicit efficient primary prevention [42,43].

Smoking levels reduced with improving wealth; so, lower levels of smoking were noted in the middle class and upper class population groups [42]. The richer the population, the lower the consumption of tobacco-based products. However, the economically-disadvantaged population groups continue to smoke “bidis”, because India has historically had no significant taxes on bidis making it more available to the poorer socioeconomic groups. Taxes should therefore be levied on all tobacco products [42,43].

Gender-based differences in smoking should be an important consideration. Women showed less interest in smoking cessation programs, because women smoking tobacco is considered culturally unacceptable in India. Most women tend to be smokeless tobacco users [42,43]. Therefore, more gender inclusivity should be practiced so that women can avail themselves of tobacco cessation services without being subjected to social stigma. Both smoking and the use of all smokeless tobacco products should be completely banned in all public places. It is therefore important to appreciate cultural barriers while promoting primary prevention strategies.

#### 2.3.3. Alcohol Use/Abuse

Regular and increasing alcohol use disrupts cardiac physiology. Binge drinking causes electro-mechanical damage, leading to arrhythmias (especially atrial fibrillation) and reduced contractility. Chronic heavy alcohol use increases the risks of atherosclerosis, ischemic disease, and dilated cardiomyopathy through mechanisms such as oxidative stress, apoptosis, mitochondrial dysfunction, and abnormal fatty acid metabolism. These changes result in myocyte damage, impaired calcium handling, and cell loss. Disease patterns are influenced by drinking behavior, nutrition, genetics, ethnicity, and gender [44].

Alcohol use is an important risk factor for the global burden of HF. In India, an estimated 340,000 deaths as well as 14.7 million disability-adjusted life years were reported in 2019 consistent with the increased use of alcohol. With increasing economic independence, the adult alcohol per capita consumption (APC) (liters) has more than doubled from 2.3 to 5.5 L in the period between 2000 and 2018. Interestingly, alcohol consumption occurs >2-fold in males as compared to females in India [45]. Additionally, the alcohol-induced disease burden appears to be more pronounced in India versus the developed western nations.

Considerable variations occur in alcohol consumption in the different states in India secondary to state and federal government policies, religious restrictions, educational levels and socioeconomic differences. Hence, alcohol bans exist currently in 6 of the 28 states and union territories due to local population preferences of alcohol use based on religious/cultural practices and the per capita state domestic product. In other states, a variety of rules to reduce consumption exist such as higher drinking age limits and more severe penalties for binge drinking. However, estimates of alcohol need to be more reliable with more robust studies in order to establish alcohol control policies against binge drinking [46]. Generating public awareness in this area seems to be a logical approach to modify this risk factor in addition to adequate government policies from a health perspective.

#### 2.3.4. Recreational Drugs and HF

The use of recreational drugs has increased to a very large extent in the last three decades in India. National surveys suggest India continues to face increasing recreational drug use [47]. Increased opioid use from 0.7% in the past to approximately 2% in the present time translates from 2 million to >22 million users. Heroin appears to be the commonest drug of abuse in India, followed by other synthetic drugs and cocaine. The Ministry of Social Justice and Empowerment has stepped up its efforts over the last decade with the institution of National Action Plan for Drug Demand Reduction in 2018 and more than 400 non-governmental organizations functioning as Integrated Rehabilitation Centers for drug addicts [47]. The role of recreational drug abuse in the etiology of toxic dilated cardiomyopathies and clinical HF should be an important consideration, as this will escalate the incidence of HF [48]. Hence, further amplification of illicit drug control by governmental policies is imperative to increase the awareness of drug abuse and its consequences and therefore to decrease the disease burden.

#### 2.3.5. Obesity as the Trigger

Obesity is a public health hazard globally and specifically affecting India in a large way. Unhealthy food habits, lack of exercise, and genetic predisposition to obesity and other risk factors such as HTN and dyslipidemia/diabetes make the development of obesity not surprising in this population. India ranks third in terms of obesity with a prevalence of 11% and 20% in adolescents and adults, respectively [49]. Such a trend should not be ignored, and campaigns at the district, state and national levels should be organized by the central and state governments to prevent obesity rising to greater epidemic levels. Therefore, early prevention should include awareness of calorie control and regular exercise regimens from childhood [50]. Such measures should be instituted at all levels in private/public school systems. The involvement of the government at the national level is very important to control such an epidemic nationwide. Further studies are needed in a more controlled fashion to achieve such efforts.

#### 2.3.6. Hypertension

The more recent health surveys such as the Fourth National Family Health Survey and Fourth District Level Health Survey/Annual Health Survey showed that HTN was more common in men (24.5%) than women (20.0%). HTH in India appears to follow a pattern of higher incidence in developed urban regions of the nation housing populations of the higher socioeconomic strata [51,52]. In a cross-sectional study of 11,657 participants from the rural areas of Trivandrum, West Godavari, and Chittoor in the southern part of India, the knowledge score, as well as awareness, showed a significant positive association with blood pressure control in hypertensive patients when adjusted for age and sex [39]. Patient education regarding disease patterns and prognosis helps improve disease management.

Protocol-based management of HTN may be a positive approach to primary prevention of disease progression [40]. However, this requires standardized management regimens in a value-based setting of practice to be successful and efficient. Cardiovascular disease is predicted to be the largest cause of death and disability in India in the current era. The prevalence of uncontrolled HTN is high in India, with 9–20% of patients achieving target BP. In order to generate guidelines applicable to the Indian environment and setting, a multidisciplinary panel comprising of cardiologists, nephrologists and general internal medicine has been set up to define the key points of HTN management [41]. Longitudinal studies in different parts of the country will be needed to tailor specificities due to inherent differences in diet and lifestyle in the different regions of India which in turn would have profound effects on the response to medical treatment.

#### 2.3.7. Dyslipidemia as a Forerunner of Ischemic Cardiomyopathy

Dyslipidemia exhibits variations in pathogenesis in different ethnicities. Hence, management strategies need to be tailored for the Indian population. The Lipid Association of India (LAI) recently published a consensus statement, which has tried to adapt the established guidelines [53]. However, the differences in the age of onset of ischemic disease, phenotypic differences possibly influenced by genotypic and cultural variations in food and socioeconomic parameters, as well as the pharmacokinetics of drug metabolism such as statins make it difficult to make it universally applicable. The side effect profile and pharmacodynamics of statins need further investigation in the Indian population. The management of dyslipidemia should be specifically tailored for Indian conditions with respect to screening and prevention because of the existing healthcare payment models, which makes it very challenging for the lower socioeconomic groups for follow-up.

#### 2.3.8. Elevated Surgical Risk Considerations in Ischemic Cardiomyopathy

South Asians have a mix of factors that make cardiac procedures more challenging. Their coronary arteries tend to be smaller, with softer non-calcified plaques in the setting of higher rates of insulin resistance, abdominal fat, and diabetes, thus increasing the pre-surgical risk. Elevated levels of lipoprotein(a) with lower levels of endothelial progenitor cells lead to faster disease progression. Renal dysfunction and more diffuse disease in the vessels make cardiac bypass surgery more complex, with slightly higher short-term risks, though the long-term outcomes can be optimal with effective post-operative care and follow-up. Post-surgery, the underlying metabolic dysfunction may reveal previously unrecognized heart failure, which can augment the risk.

The affordability of patients undergoing advanced cardiac surgical therapies such as the implantation of a left ventricular assist device or cardiac transplantation still remains a challenge for end stage heart failure patients. The 2024 NITI Aayog mission document would probably define the framework for a national program to make India self-reliant in producing affordable high-quality ventricular assist devices, with the potential to transform outcomes for thousands of patients.

### 2.4. Current Risk Assessment Resources and Risk Prediction Models

Risk assessment for heart failure (HF) in South Asian populations is particularly challenging because standard Western-derived models for assessing risk for cardiovascular disease frequently underestimate the risk in this group. PCP-HF (Pooled Cohort Equations to Prevent Heart Failure) is a primary multivariable model used to predict the 10-year HF risk [54]. However, research from the MASALA study shows that, while it can categorize risk, it may not fully capture the unique South Asian risk profile. Atherosclerotic Cardiovascular Disease (ASCVD) Pooled Cohort Equations (PCE), though primarily used for atherosclerosis, are often used as a baseline for South Asians, because being of South Asian ethnicity is being recognized as a “risk-enhancing factor”, which should prompt earlier or more aggressive intervention [55]. The PCE usually underestimates the 10-year risk of ASCVD in individuals of South Asian descent by up to two-fold. Because the PCE was derived from populations excluding South Asians, it often underrepresents the higher prevalence of diabetes, hypertension, and abdominal obesity in this group. The QRISK3 developed in the UK, includes South Asian ethnicity as a specific variable, applying a multiplication factor (typically 1.3 to 1.7) to account for the higher inherent risk [55]. SCORE2-ASIA is a newer regional model introduced by the European Society of Cardiology (ESC), specifically for Asian populations, aimed at improving calibration compared to previous European models [56].

South Asian Indians have a two-fold higher risk of heart disease compared to Europeans, often developing coronary artery disease 10 years earlier. The risk of heart failure (HF) risk is elevated due to unique factors including a high diabetes rates, visceral fat despite low BMI, and high lipoprotein(a), which is often underestimated by standard risk calculators. The linear statistical models currently used in South Asian Indians need modification and validation.

The utility of AI-driven technologies to predict cardiovascular risk is being investigated with progress noted in the current literature. AI-driven machine learning (ML) models such as random forest and XGBoost appear to significantly improve cardiovascular disease risk assessment in South Asians by achieving up to 93.8% accuracy, outperforming traditional tools that often underestimate the risk in this population. These systems analyze multi-dimensional data, including genetic markers such as the 25-bp deletion in the MYBPC3 gene, factors affecting lifestyle, and ECG, to predict high-risk premature cardiovascular disease at younger ages. Extreme gradient boosting (XGBoost), random forest (RF), and logistic regression (LR) are highly effective in identifying hypertension and heart failure risk, often outperforming traditional statistical methods [57]. The use of machine learning models used in the Kerala Atrial fibrillation registry yielded a good discriminatory power of predictive performance in the internal validation with a receiver operator characteristic curve of (AUC) 0.821 using the random forest method, but this did not hold up in the external validation with the light gradient boosting machine learning model showing the best predictive performance with AUC 0.670 in the external validation [58]. Such discrepancies noted on external validation dictate the need for more robust models to be tested and validated in different datasets. The combined use of traditional linear statistical methods and machine-learning approaches in developing risk prediction models for HF, including the identification of novel risk factors, should form the basis for future research.

### 2.5. Existing Challenges in Generating Robust Risk Prediction Models in the South Asian Indian Population

There is currently a lack of representative data. The scarcity of population-specific data arises because most of the existing HF and cardiovascular (CVD) data are derived from Caucasian U.S. White or European populations, with missing data on the resident Indian population living in the subcontinent. No currently available risk models are based on data from resident Indians, who have a significantly higher CVD risk than Western populations. Additionally, inadequate subgroup disaggregation exists. South Asians are often grouped together, masking significant heterogeneity in risk factor prevalence and disease incidence between subgroups such as Indians, Pakistanis, and Bangladeshis.

At the genetic and molecular levels, there is molecular underrepresentation. Genome-wide and metabolomic studies are primarily driven by samples of European ancestry, which may not capture the unique molecular risk profiles in South Asians. Some of the yet unidentified causal factors include premature and more severe disease, and traditional risk factors do not fully explain this burden, suggesting the existence of yet-unidentified population-specific genetic or gene-modulating factors. The lipid abnormalities need to be investigated, as to whether the high risk at lower LDL-C levels is due to a higher particle burden or complex metabolic results, such as insulin resistance and elevated triglycerides.

The unique phenotypic and clinical presentations need special attention. The premature onset of heart failure often manifests 10 to 15 years earlier in South Asians compared to other groups; however, standard screening thresholds are not optimized for this younger onset. The paradox of low BMI and the metabolic syndrome make the use of the BMI cutoffs based on the Caucasian race inappropriate. Another important difference in this population is the heart failure with preserved ejection fraction (HFpEF) heterogeneity, which develops at a younger age and in leaner individuals among South Asian Indians, a pattern that is not well-captured by standard models.

**Figure 2:**
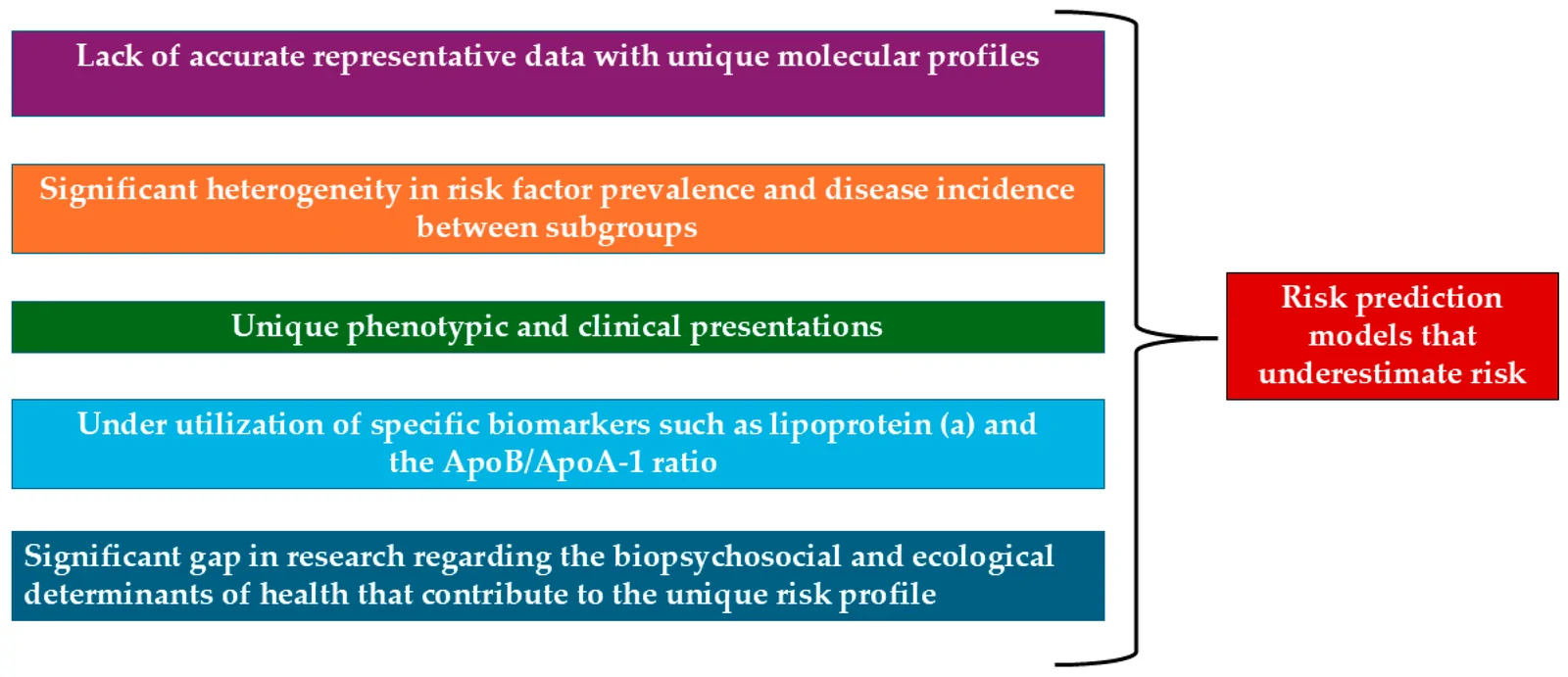
Existing challenges in generating robust risk prediction models for heart failure.

There is underutilization and a lack of integration of non-traditional biomarkers such as lipoprotein(a) and the ApoB/ApoA-1 ratio, which are important for risk stratification significant for South Asians but are rarely integrated into standard risk scores. Coronary artery calcium (CAC) is a specific marker for subclinical atherosclerosis in this group, which is also often unaccounted for in primary prevention stratification. There is a significant gap in research regarding the biopsychosocial and ecological determinants of health that contribute to the unique risk profile of South Asian Indians, which also impacts early detection and the effectiveness of predictive models in real-world settings. A summary of the challenges in HF prediction in South Asian Indians is shown in Figure 2.

### 2.6. Differences and Similarities in Disease Progression in Indians Versus the Global Indian Diaspora

The heart failure (HF) risk among Indians varies substantially between those living in India and members of the global Indian diaspora. Although both groups share underlying genetic predispositions, their environmental exposures, lifestyle patterns, healthcare access, and sociocultural contexts diverge in ways that meaningfully shape the HF incidence, phenotype, and outcomes.

Indians living in India experience HF at younger ages and with higher mortality than most populations worldwide. Several factors contribute to this pattern. First, India carries a heavy burden of traditional cardiovascular risk factors, with hypertension, diabetes, and coronary artery disease often developing earlier and progressing more aggressively. Rapid urbanization, dietary transitions toward calorie-dense foods, high rates of physical inactivity, and persistent exposure to air pollution amplify metabolic and vascular stress. Visceral adiposity despite a normal BMI, a hallmark of South Asian physiology, is widespread and intersects with high rates of insulin resistance and type 2 diabetes. These biological vulnerabilities are compounded by socioeconomic disparities, limited access to preventive care, delayed diagnosis, and variability in treatment quality across urban and rural settings. As a result, HF in India often presents late, with advanced symptoms, reduced ejection fraction, and poorer survival.

In contrast, the global Indian diaspora particularly in North America, Europe, and the Middle East experiences a different HF risk profile shaped by migration, acculturation, and improved healthcare access. While diaspora Indians retain the same genetic and metabolic predispositions, their environment often modifies the disease expression. Access to structured preventive care, routine screening, and evidence-based cardiovascular therapies is generally better than in India, enabling earlier detection of hypertension, diabetes, and coronary disease. This can delay HF onset and improve outcomes. However, diaspora populations also face unique challenges. Acculturation frequently leads to the adoption of Western dietary patterns high in saturated fats and processed foods, contributing to obesity and worsening the metabolic risk. Sedentary lifestyles, occupational stress, and psychosocial pressures related to immigration and socioeconomic mobility further elevate cardiovascular strain.

Interestingly, HF phenotypes differ between the two groups. Indians in India more commonly present with ischemic cardiomyopathy driven by premature coronary artery disease, whereas diaspora Indians show a higher prevalence of HF with preserved ejection fraction (HFpEF), reflecting obesity, hypertension, and metabolic syndrome associated with lifestyle changes after migration. In countries with strong primary care systems, diaspora Indians may also benefit from earlier initiation of guideline-directed medical therapy, cardiac rehabilitation, and advanced interventions such as revascularization or device therapy, improving their long-term survival [59].

Social determinants of health create additional divergence. In India, disparities in education, income, and healthcare infrastructure strongly influence the HF risk and outcomes. In diaspora communities, socioeconomic gradients persist but are shaped by immigration status, cultural integration, and access to healthcare. Cultural dietary practices, family structures, and health-seeking behaviors also evolve differently across settings, influencing risk trajectories.

**Figure 3:**
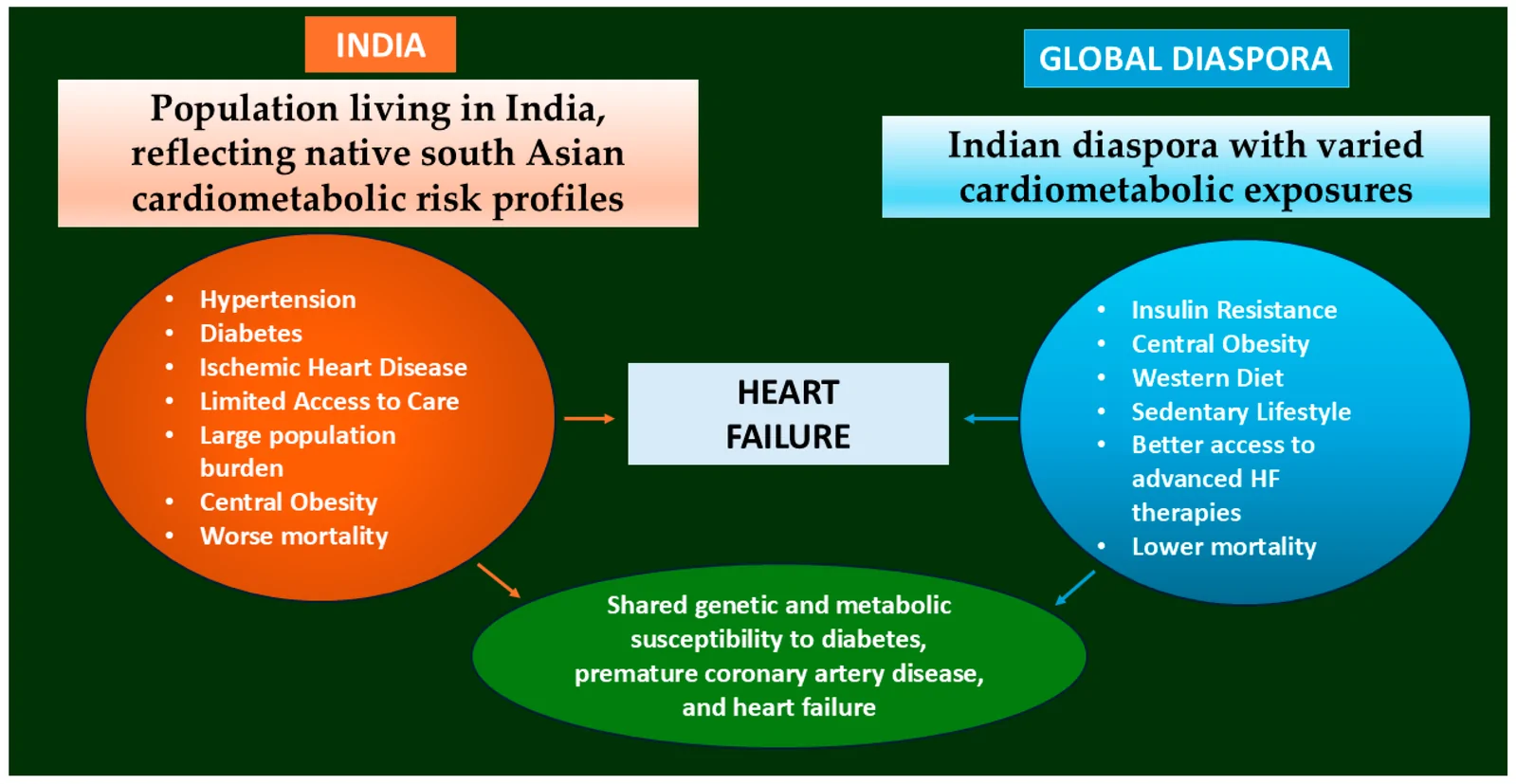
HF risk in Indians versus global diaspora.

Despite these differences, both groups share a common challenge, i.e., traditional cardiovascular risk models consistently underestimate the HF risk in South Asians (Figure 3). Precision approaches including AI-driven prediction tools may help capture the interplay of genetics, metabolic traits, and environment across both populations.

## 3. Discussion

### 3.1. Evaluation of Existing Risk Prediction Models for HF in South Asians

The existing heart failure (HF) risk prediction in South Asians is largely borrowed from models developed in non-South Asian populations, with very limited South Asian-specific tools. The best-studied application is in the MASALA cohort, which used the Pooled Cohort Equations to Prevent Heart Failure (PCE-HF) rather than specific South Asian models [60]. The PCE-HF was derived from large predominantly White and Black U.S. cohorts to estimate the 10-year incident HF risk using age, sex, blood pressure, diabetes, BMI, coronary artery disease, and other clinical variables. It was not originally calibrated for South Asians. In MASALA, investigators calculated the 10-year HF risk using PCE-HF in 1159 South Asian Americans. About 40% had low (<1%), 37% intermediate (1–5%), and 24% high (≥5%) predicted HF risk, with strong associations between higher risk and diabetes, hypertension, BMI, and coronary artery calcium. Social determinants such as income, education, and birthplace also stratified the risk, underscoring the importance of non-biologic factors. The strengths of the analyses include a validated framework, practicality, and the first systematic look at HF risk distribution in south Asians. The PCE-HF is a rigorously derived multivariable model with good performance in its derivation cohorts. This model uses routinely available clinical variables, making it easy to implement in South Asian clinical settings and diaspora cohorts. The use of PCE-HF in MASALA provides valuable insight into how many South Asians fall into intermediate/high HF risk categories and which factors drive that risk. However, there are limitations. This model is not South Asian-specific. The model was not derived or recalibrated using South Asian data, and hence, it may misestimate the absolute risk due to differences in baseline incidence, body composition, and metabolic profile. There would be underrepresentation of unique traits, because the visceral adiposity at a lower BMI, earlier diabetes onset, and distinct lipid patterns are not explicitly modeled. Additionally, there has been no India-based validation. The PCE-HF has been evaluated in South Asian Americans, but not in large community-based cohorts in India, where environmental exposures and healthcare access differ substantially.

Other cardiovascular risk models such as Framingham, QRISK, and ASCVD Pooled Cohort Equations (for atherosclerotic events) are widely used in South Asians for coronary and stroke risk, but they are not HF-specific and often underestimate the risk in South Asians [61]. Some regional tools (e.g., QRISK3 in the UK) include “South Asian ethnicity” as a risk modifier but still focus on atherosclerotic events rather than HF and are calibrated to local populations, not populations living in the Indian subcontinent.

Hence, only one major HF-specific model (PCE-HF) has been applied to South Asians in MASALA, showing a high predicted HF risk and clear associations with diabetes, hypertension, BMI, and coronary artery calcium, but no HF risk model derived in South Asian cohorts (India-based or multi-country South Asian datasets) exists. This has major implications, because there has been no recalibration of PCE-HF or other HF scores to South Asian incidence rates, phenotypes (HFpEF vs. HFrEF), or genetic/epigenetic factors, and there is limited integration of social determinants and environmental exposures (air pollution, tobacco forms, diet, and urbanization) that are highly relevant in the populations living in India. Therefore, the current HF risk prediction in South Asians relies on extrapolation from non-South Asian models (primarily PCE-HF), with only one major diaspora validation (MASALA) and no India-specific derivation or calibration, making this a key area for future research and AI-driven population-specific modeling.

### 3.2. Future Perspectives Using AI Driven Approaches

Future efforts to develop heart failure (HF) risk-prediction models for South Asian Indians must move beyond traditional risk factors to better capture this population’s uniquely high and premature disease burden, as denoted in the graphical abstract. Existing calculators often underestimate the risk, because South Asians were historically underrepresented in the cohorts used to build them.

A major priority is integrating ancestry-specific genetic data. Developing and validating genome-wide polygenic scores tailored to South Asian ancestry could help identify high-risk individuals independent of conventional factors. Incorporating markers such as lipoprotein(a) and other gene-modulating traits common in South Asian communities may further strengthen the prediction.

AI-driven approaches including machine learning and deep learning can leverage large datasets spanning biomarkers, imaging, metabolomics, epigenomics, and proteomics to uncover complex risk patterns often missed in models derived from predominantly Caucasian populations. Real-world data from electronic medical records and wearable devices may also enable dynamic lifetime risk estimation.

Models should incorporate population-specific risk enhancers, including South Asian-specific BMI cutoffs and measures of visceral adiposity, given the higher HF risk at lower BMI thresholds. Subclinical imaging markers such as coronary artery calcium scores and carotid intima-media thickness can refine the risk in borderline categories. Sociocultural factors such as dietary patterns, environmental exposures, and social determinants must also be integrated.

**Figure 4:**
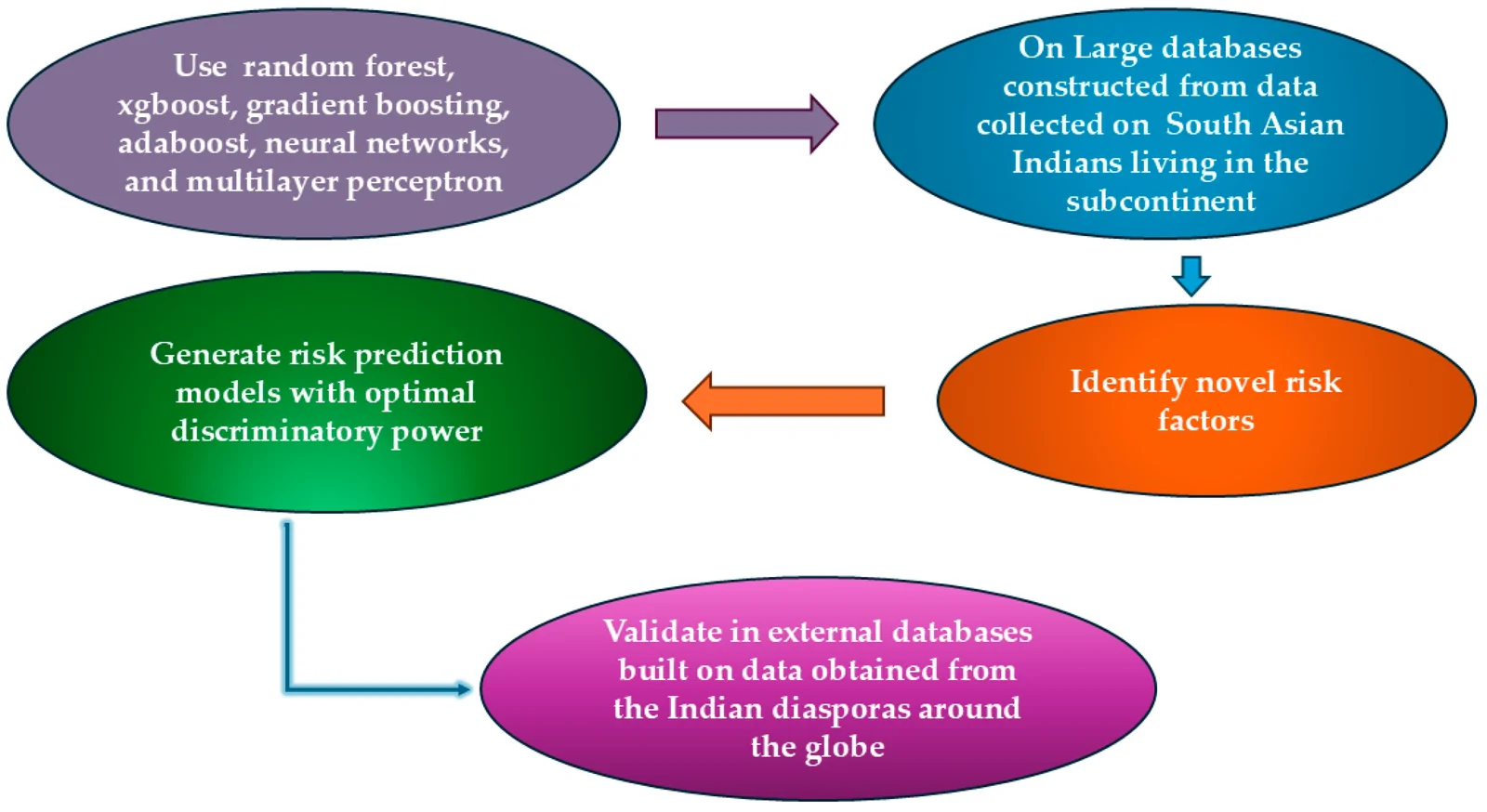
Use of AI-driven strategies for risk prediction for HF in South Asian Indians.

AI will play a central role in advancing prediction. Noise-adapted AI-ECG algorithms using single-lead or wearable ECGs have already shown markedly higher HF risk detection [62]. Multimodal deep-learning models that analyze clinical data alongside scanned paper ECGs may be especially valuable in regions where digital ECGs are less common. Ensemble methods such as random forest, XGBoost, and CatBoost, along with neural networks and molecular AI tools, can further enhance early detection. The use of AI on large databases to develop better prediction models is shown in Figure 4.

HF prediction models for South Asian Indians must reflect their distinct genetic, lifestyle, and clinical profiles, due to their 10–15-year earlier onset of cardiovascular disease.

### 3.3. Strengths

This study has a structured search strategy and addresses a major evidence gap, because definitive clinical trials addressing HF outcomes in this population remain limited. The manuscript highlights the upstream HF risk drivers and outlines actionable prevention insights such as relevant lifestyle, tobacco, alcohol, and obesity-related interventions with implications for public health planning.

### 3.4. Limitations

Although the search strategy is defined, the review does not include risk-of-bias assessment, evidence grading, or meta-analytic synthesis because of the lack of definitive studies with HF specific models currently developed for South Asians.

## 4. Conclusions

South Asian Indians face an unusually high and early risk of heart failure. Although they represent a quarter of the world’s population, they bear nearly 60% of global heart disease, often developing myocardial infarction and heart failure a decade earlier than other groups. This early onset, rising mortality, and frequent underestimation by standard risk models reflect a distinct metabolic profile consisting of visceral adiposity with normal BMI, insulin resistance, high diabetes incidence, and atypical lipids. These biological factors intersect with genetics and social determinants. AI-driven tools may enable more accurate tailored HF risk prediction, especially in the setting of definitive clinical trials. South Asians currently rely on non-South Asian HF risk models, with PCE-HF being the only one applied but not calibrated for their unique risk profile. There is an urgent need for South Asian-specific HF risk prediction, ideally integrating genomics, visceral adiposity, early metabolic dysfunction, and environmental exposures.

## Data Availability

Data use to generate this abstract will be available on request.

## Abbreviations

The following abbreviations are used in this manuscript:

HF	Heart Failure
ASCVD	Atherosclerotic Cardio Vascular Disease
PCE	Pooled Cohort Equations
MASALA	Mediators of Atherosclerosis in South Asians Living in America
CAC	Coronary Artery Calcium
PODOSA	Prevention of Diabetes and Obesity in South Asians
SAHELI	South Asian Healthy Lifestyle Intervention
IGVC	Indian Genome Variation Consortium
GWAS	Genome Wide Association Study
CSIR	Council for Scientific and Industrial Research
